# Poster Session II - A255 *IL1B*-RS1143634 ASSOCIATED TO NONRESPONSE TO INFLIXIMAB IN ULCERATIVE COLITIS TREATMENT

**DOI:** 10.1093/jcag/gwaf042.255

**Published:** 2026-02-13

**Authors:** L Tessier, G Boucher, A Lavoie, A Michaud-Herbst, J D Rioux, K Tremblay

**Affiliations:** Pharmacology–Physiology Department, Université de Sherbrooke, Saguenay, QC, Canada; Institut De Cardiologie de Montreal, Montreal, QC, Canada; Pharmacy Department, Centre Intégré Universitaire de Santé et de Services Sociaux du Saguenay–Lac-Saint-Jean (CIUSSS-SLSJ, University Hospital), Saguenay, QC, Canada; Gastroenterology Department, Centre Intégré Universitaire de Santé et de Services Sociaux du Saguenay–Lac-Saint-Jean (CIUSSS-SLSJ, University Hospital), Saguenay, QC, Canada; Universite de Montreal Faculte de Medecine, Montreal, QC, Canada; Pharmacology–Physiology Department, Université de Sherbrooke, Saguenay, QC, Canada

## Abstract

**Background:**

Molecular targeted therapies (MTT), such as anti-TNFs (infliximab, adalimumab and golimumab), are used to achieve remission in moderate to severe ulcerative colitis (UC) patients. We observe a high variability in treatment response to these therapies which is influenced by multiple factors such as genetics. Indeed, 102 variants are associated with response phenotype in inflammatory bowel disease (IBD) in the literature, but none has strong enough evidence to be used to predict response.

**Aims:**

Therefore, the aim of this study is to perform a genetic association study of these candidate pharmacogenetic variants.

**Methods:**

A cohort of 101 people using MTT for UC in Saguenay–Lac-St-Jean was recruited. The treatment response was evaluated retrospectively and patient’s whole genome was genotyped. The variants list obtained by literature review was validated by a burden test comparing responders and non-responders. Once the list was validated, the association of the variants was evaluated in the recruited cohort. Results from the significantly associated variants were compared with other IBD studies in literature.

**Results:**

The variants list was validated only for the infliximab group (n = 40, p = 0.029) and not for the anti-TNF combined group (n = 67, p = 0.770). For the infliximab group, the variant *IL1B*-rs1143634 was associated with nonresponse (p = 0.01). Additionally, a literature review revealed two other studies had verified the associated variant in IBD and the association was maintained after the combination of these three studies (OR = 1.97 [1.29-3.01], p = 0.002).

**Conclusions:**

The association of *IL1B*-rs1143634 increases its interest to better understand nonresponse to infliximab. Further replication and functional studies are necessary for this variant to become a potential clinical marker.

**Funding Agencies:**

CCC, CIHRFRQS, Fondation de ma vie, Faculté de médecine et des sciences de la santé de Université de Sherbrooke

